# Genetic Association in the Maintenance of the Mitochondrial Microenvironment and Sperm Capacity

**DOI:** 10.1155/2021/5561395

**Published:** 2021-09-04

**Authors:** Hwang I. S. Thomas, Ying-Shiuan Chen, Ching-Han Hung, Dilip Bhargava Sreerangaraja Urs, Tien-Ling Liao, Yen-Chun Lai, Katerina Komrskova, Pavla Postlerová, Yung-Feng Lin, Shu-Huei Kao

**Affiliations:** ^1^Division of Urology, Department of Surgery, Shin Kong Wu Ho-Su Memorial Hospital, Taipei, Taiwan; ^2^Department of Urology, College of Medicine, Taipei Medical University, Taipei, Taiwan; ^3^Department of Urology, School of Medicine, Fu-Jen Catholic University, New Taipei, Taiwan; ^4^School of Medical Laboratory Science and Biotechnology, College of Medical Science and Technology, Taipei Medical University, Taipei, Taiwan; ^5^Department of Gynecology and Obstetrics, Taipei City Hospital Ren-Ai Branch, Taipei, Taiwan; ^6^Ph.D. Program in Medical Biotechnology, College of Medical Science and Technology, Taipei Medical University, Taipei, Taiwan; ^7^Laboratory of Reproductive Biology, Institute of Biotechnology of the Czech Academy of Sciences (BIOCEV), Vestec, Czech Republic; ^8^Department of Zoology, Faculty of Science, Charles University, Prague, Czech Republic; ^9^Department of Veterinary Sciences, Faculty of Agrobiology, Food and Natural Resources, University of Life Sciences Prague, Prague, Czech Republic; ^10^Center for Reproductive Medicine and Sciences, Taipei Medical University Hospital, Taipei, Taiwan

## Abstract

Sperm motility is one of the major determinants of male fertility. Since sperm need a great deal of energy to support their fast movement by active metabolism, they are thus extremely vulnerable to oxidative damage by the reactive oxygen species (ROS) and other free radicals generated as byproducts in the electron transport chain. The present study is aimed at understanding the impact of a mitochondrial oxidizing/reducing microenvironment in the etiopathology of male infertility. We detected the mitochondrial DNA (mtDNA) 4,977 bp deletion in human sperm. We examined the gene mutation of ATP synthase 6 (*ATPase6* m.T8993G) in ATP generation, the gene polymorphisms of uncoupling protein 2 (*UCP2*, G-866A) in the uncoupling of oxidative phosphorylation, the role of genes such as manganese superoxide dismutase (*MnSOD*, C47T) and catalase (*CAT*, C-262T) in the scavenging system in neutralizing reactive oxygen species, and the role of human 8-oxoguanine DNA glycosylase (*hOGG1*, C1245G) in 8-hydroxy-2′-deoxyguanosine (8-OHdG) repair. We found that the sperm with higher motility were found to have a higher mitochondrial membrane potential and mitochondrial bioenergetics. The genotype frequencies of *UCP2* G-866A, *MnSOD* C47T, and *CAT* C-262T were found to be significantly different among the fertile subjects, the infertile subjects with more than 50% motility, and the infertile subjects with less than 50% motility. A higher prevalence of the mtDNA 4,977 bp deletion was found in the subjects with impaired sperm motility and fertility. Furthermore, we found that there were significant differences between the occurrences of the mtDNA 4,977 bp deletion and *MnSOD* (C47T) and *hOGG1* (C1245G). In conclusion, the maintenance of the mitochondrial redox microenvironment and genome integrity is an important issue in sperm motility and fertility.

## 1. Introduction

Male infertility is a growing problem that affects 30% of infertile human couples due to a decline in sperm counts and rise in testicular and sperm anomalies. The evaluation of male-factor infertility has become more important and informative since new diagnostic techniques and therapeutic options have become available. Poor sperm motility has been considered as one of the major causes of male infertility [1]. It is highly probable that the respiratory dysfunction of mitochondria causes a decline in motility [2]. However, there remains a group of these subfertile men in whom routine semen analysis results are within normal values and who are classified as having unexplained male infertility. The presence of antisperm antibodies, sperm DNA damage, and oxidative stress has been suggested to contribute to unexplained male infertility [3].

In mammalian germ cells, reactive oxygen species (ROS) have been shown to be required for sperm maturation, differentiation, capacitation, acrosomal reaction, zona pellucida binding, and oocyte fusion [4]. Notably, ROS levels in semen are higher in infertile males [5, 6]. Excessive generation of ROS was found to be associated with idiopathic male infertility and sperm apoptosis [7]. In addition to the conventional causes for male infertility, cryptorchidism, infections, obstructive lesions, cystic fibrosis, trauma, and tumors have been identified to be associated with oxidative stress [8, 9]. Oxidative stress is represented as a major cause of male fertility in more than 40% of patients revealing evidences of oxidative attack, resulting in high levels of lipid peroxidation and oxidative DNA damage. Extraordinary levels of deleterious ROS lead to DNA damages and fragmentation, motility impairment, mitochondrial dysfunction, and cell apoptosis in human sperm [10–13]. It is important to point out that the oxidative DNA adduct, 8-hydroxy-2′-deoxyguanosine (8-OHdG) is highly mutagenic and might elicit *de novo* mutations during spermatogenesis [14]. More than 9000 genomic lesions in the human sperm genome have been found as highly vulnerable to oxidative attack in human sperm [15]. Oxidative stress-mediated DNA damage may be the etiology for repeated assisted reproductive technology failures [16, 17].

Mitochondrial ATP generation increases sperm linear motility that might have an impact on the *in vivo* transfer of sperm from the uterus to the oviduct [18]. There is reason to believe that sperm mitochondria are one of the major targets of attack by ROS, and mitochondria in particular have been identified as a major source of ROS through electron leakage from mitochondrial respiratory Complexes I and III [19, 20]. The deleterious ROS are usually disposed of by the coordinated functioning of enzymatic antioxidants, but a certain fraction of them may escape the antioxidant defense system and cause transient or permanent DNA damages [17, 21]. Thus, we hypothesized that redox control in the mitochondrial microenvironment is essential for proper sperm motility and fertility. In this study, we investigated the polymorphisms and allele frequencies of these genes contributing to the maintenance of mitochondrial energy generation and oxidative scavenging capacity (Table 1). We examined the gene mutation of ATP synthase 6 (*ATPase6* m.T8993G) in ATP generation, the gene polymorphisms of uncoupling protein 2 (*UCP2*, G-866A) in the uncoupling of oxidative phosphorylation (OXPHOS), the role of genes such as manganese superoxide dismutase (*MnSOD*, C47T) and catalase (*CAT*, C-262T) in the scavenging system in neutralizing ROS, and the role of human 8-oxoguanine DNA glycosylase (*hOGG1*, C1245G) in 8-OHdG repair. We also analyzed the association between the occurrence of an mtDNA common deletion (4,977 bp deletion) and the polymorphisms of these genes. We found that maintenance of the mitochondrial redox microenvironment is an important issue in genome integrity, sperm motility, and fertility.

## 2. Materials and Methods

### 2.1. Semen Collection and Assessment of Sperm Motility Characteristics

We collected 220 semen samples from 58 healthy donors who had normal semen characteristics and from 162 infertile or subfertile males at Hsin Kong Wu Ho-Su Memorial Hospital and Taipei City Hospital Ren-Ai Branch. This study was performed according to the tenets of the Declaration of Helsinki for research involving human subjects. The protocol was approved by the Institutional Review Board/Ethics Committee of Hsin Kong Wu Ho-Su Memorial Hospital and Taipei City Hospital Ren-Ai Branch. After informed patient consent was obtained, the semen samples were collected. All of the semen samples were obtained by masturbation after 3–4 days of abstinence. After liquefaction, the characteristics of sperm motility were examined using a computer-assisted semen analyzer (CASA; HTM-2000 motility analyzer; Hamilton Thorn Research, Danvers, MA). Leukospermia and viscous semen samples were excluded from this study.

### 2.2. Ficoll-Paque Fractionation and Sperm Preparation

To avoid the contamination of sperm by other types of cells such as lymphocytes and epithelial cells, we removed the contaminant cells with Ficoll-Paque (Pharmacia Biotech AB, Uppsala, Sweden) separation before DNA extraction and flow cytometric analysis. Sperm were separated from seminal plasma by centrifugation at 300 × *g* for 10 min at 25°C. The sperm pellet was resuspended in phosphate-buffered saline (PBS; Dulbecco Oxoid, UniPath Ltd., Hants, UK; pH 7.3), and the final sperm count was adjusted to 2 ~ 4 × 10^8^ sperm/ml. An aliquot of the suspension was layered on the top of a tube containing 2 ml of 60% and 80% Percoll gradient in Ham's F10 medium and was incubated at 37°C for 90 min. After incubation, the sperm in the different Percoll gradients were collected and washed with PBS before centrifugation at 300 × *g* for 10 min.

### 2.3. Mitochondrial Membrane Potential in the H_2_O_2_-Treated Human Sperm

To visualize the changes in the sperm mitochondrial membrane potential under oxidative stress, sperm were treated with 100 *μ*M H_2_O_2_ and then stained for 10 min with 10 *μ*M JC-1 (Molecular Probes, Eugene, OR) at 37°C. The dye at lower mitochondrial concentrations with lower ∆Ψ forms a green fluorescent monomer with emissions at 530 nm, but at higher concentrations, it forms red fluorescent aggregates with emissions at 590 nm. All analyses were performed by confocal fluorescence microscopy (Leica TCS SP5, Leica Microsystems CMS GmbH, Mannheim, Germany) and flow cytometry (FACScan, Becton Dickson, San Jose, CA). Confocal fluorescent images were captured using the Leica SP5 confocal microscope fitted with an Apochromat 63x/1.4 NA immersion objective and with three lasers (argon, 488 nm; diode, 405 nm). In addition, a minimum of 3 × 10^4^ cells per sample were analyzed by flow cytometry. The relative proportions of cells within different areas of the fluorescence profile were quantified with the LYSYS II software program (Becton Dickson).

### 2.4. Mitochondrial Bioenergetics

The oxygen consumption from extracellular flux analysis of oxygen consumption of sperm was measured using the Seahorse XF extracellular flux analyzers (Seahorse Bioscience, North Billerica, MA). Fresh 1 × 10^7^ sperm samples were placed in 24-well analysis plates, and the volume was adjusted to 0.5 ml. The oxidative phosphorylation capacity of sperm was analyzed after the condition of sperm was equilibrated for 20 minutes. Three chemicals were sequentially injected into the assay medium including 5 *μ*M oligomycin (Complex V inhibitor) at the time of 32 minutes, 1 *μ*M trifluorocarbonylcyanide phenylhydrazone (FCCP, mitochondrial uncoupler) at the time of 56 minutes, and 3 *μ*M rotenone (Complex I inhibitor) at the time of 88 minutes. Results of sperm oxygen consumption rate (OCR) were calibrated with sperm number in each well and analyzed by the Seahorse XF24 software.

### 2.5. DNA Extraction from Human Sperm

Before sperm DNA extraction, an aliquot of 3‐5 × 10^7^ sperm was treated with osmotic shock. Sperm were incubated in 15 ml of 50 mM Tris-HCl buffer (pH 6.8) at 8°C for 20 min to lyse the contaminated cells. Sperm cells, which were resistant to this treatment, were then subjected to DNA extraction according to the method described previously [5]. After digestion at 56°C for 2 h in 1.5 ml lysis buffer, the lysate was extracted once each with phenol, phenol/chloroform, and chloroform in succession. The aqueous layers were pooled and precipitated with isopropanol (1 : 1, *v*/*v*) and one-tenth volume of 3 M sodium acetate (pH 5.6), and incubated at -20°C overnight. The sperm DNA was finally dissolved in 10 mM Tris-HCl buffer (pH 8.5).

### 2.6. Detection of the 4,977 bp MtDNA Deletion in Human Sperm

We performed PCR to analyze the occurrence of 4,977 bp mtDNA deletion using primer pairs L8150 (8150-8169) and H13845 (13845-13826). The nucleotide sequences of the primer pairs used are listed in Table 2. The desired segment was amplified from approximately 100 ng of each DNA sample in a 50 *μ*l reaction mixture containing 200 *μ*M of each dNTP, 0.6 *μ*M of primers, 1 unit of Taq DNA polymerase (PerkinElmer Life Science, Inc. Boston, MA), 50 mM KCl, 1.5 mM MgCl_2_, and 10 mM Tris-HCl (pH 8.3). PCR was carried out for 25 cycles in a DNA thermal cycler (Model 9600, PerkinElmer). Amplified nucleotide fragments of 719 bp were separated by electrophoresis on 1.5% agarose gels and detected after staining with 0.5 mg/ml ethidium bromide.

### 2.7. Immunolocalization of 8-OHdG in Human Sperm

Intracellular localization of 8-OHdG was performed with paraformaldehyde-fixed sperm on slides. After two washes with PBS, sperm were treated with cold methanol for 3–5 minutes, washed with PBS, and then incubated for 1 hour at room temperature with 2% bovine serum albumin to prevent nonspecific binding of antibodies. Sperm then were incubated for 24 hours with anti-8-OHdG mouse monoclonal antibody anti-8-OHdG (SC66036 Santa Cruz® Biotechnology, CA, USA), followed by washing with PBS and treatment with Alexa Fluor® 647 (ab150115, Abcam, Cambridge, UK). To visualize sperm head, cells were stained for 15 minutes with 1 *μ*g/ml DAPI (Molecular Probes, Eugene, OR). This probe shows blue fluorescence.

### 2.8. Genotyping of the Polymorphisms

The point mutation of *ATPase6* (m.T8993G) and the SNPs of *UCP2* (G-866A, rs659366), *MnSOD* (C47T, rs4880), *CAT* (C-262T, rs1001179), and *hOGG1* (C1245G, rs1052133) were analyzed by polymerase chain reaction (PCR) and restriction fragment length polymorphism (RFLP; MJ Research, MA) analysis. The PCR-RFLP assay consisted of primer pairs for PCR amplification and restriction enzymes for digestion; the size products are shown in Table 2. The genotypes of the PCR products were confirmed by DNA sequence analysis. The primers with desired DNA sequences were chemically synthesized by Roche Molecular Systems Inc. (Branchburg, NJ, USA).

### 2.9. Statistical Analysis

The significance of the correlations among the gene polymorphisms, sperm motility, and fertility was determined with the Chi-squared test using IBM SPSS Statistics 19 (IBM. Armonk, NY). *p* values less than 0.05 were considered significant.

## 3. Results

### 3.1. Oxidative Stress Affects the Mitochondrial Membrane Potential (Δ*ψm*) of Human Sperm

**Figure 1:**
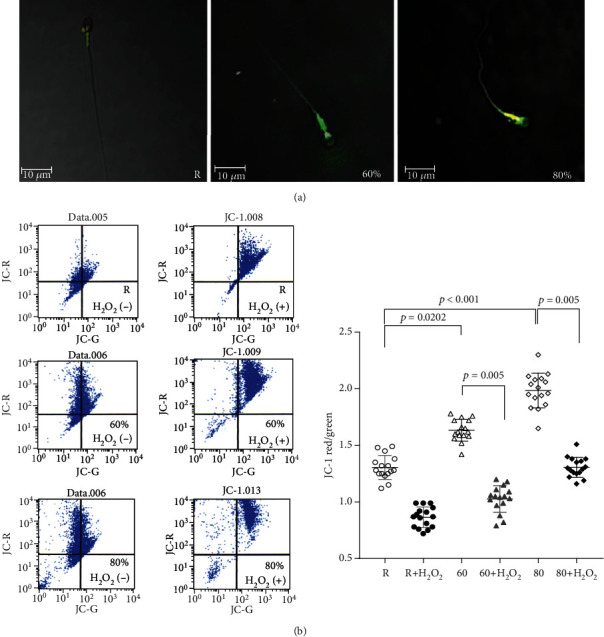
Oxidative insult affects the mitochondrial membrane potential (Δ*ψm*) of human sperm. By using JC-1, we analyzed the changes in the mitochondrial membrane potential of the Percoll-fractionated human sperm by flow cytometry and confocal microscopy. Three sperm fractions were obtained according to their motility and categorized into the 80% Percoll fraction (80%), 60% Percoll fraction (60%), and the residual fractions (R). (a) Fluorescent images of the JC-1-stained human sperm. Illustration of JC-1 accumulating preferentially in the mitochondria, existing as a green fluorescent monomer at low membrane potentials and as red-orange fluorescent aggregates at high membrane potentials. The JC-1 aggregate staining of the mitochondria was visualized in the sperm from the 80% Percoll fraction. (b) Dot plot of the mitochondrial membrane potential of human sperm by flow cytometry was represented. Flow data were assessed and expressed as the ratio of red fluorescent intensity versus green fluorescent intensity. The good motile sperm were demonstrated to harbor a higher Δ*ψm*. All three sperm fractions exposed to hydrogen peroxide lost their Δ*ψm*. Data are presented as the mean ± standard deviation (SD). ^∗∗^*p* < 0.01 compared with the control group.

Following Percoll fractionation, three sperm fractions were obtained according to their motility and categorized into the 80% Percoll fraction (80%), 60% Percoll fraction (60%), and the residual fraction (R). The JC-1 aggregate staining of the mitochondria was visualized in the sperm from the 80% Percoll fraction (Figure 1(a)). The results showed a significant positive correlation between the changes in membrane potential and sperm motility (*n* = 35, *p* < 0.01). The sperm with higher motility were found to have a higher Δ*ψm* (Figures 1(a) and 1(b)). Treatment of H_2_O_2_ was found to decrease the Δ*ψm* of sperm in all three fractions (Figure 1(b)).

### 3.2. Oxidative Stress Decreased Sperm Bioenergetics

**Figure 2:**
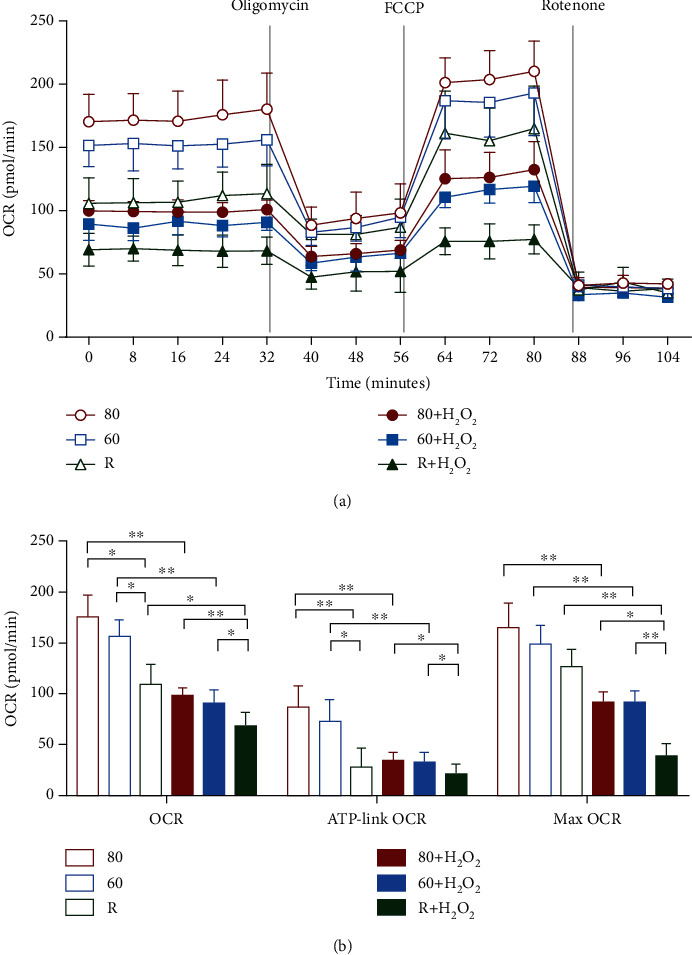
Oxidative stress reduced mitochondrial bioenergetics of human sperm. (a) Mitochondrial respiration as indicators of cellular bioenergetics were assessed using a Seahorse XF24 metabolic flux analyzer. Representative data shows the real-time oxygen consumption rate (OCR) of 1 × 10^7^ sperm with or without H_2_O_2_ treatment. Dotted lines indicate time of sequential addition of 5 *μ*M oligomycin (the ATP synthase inhibitor), 3 *μ*M FCCP (mitochondria uncoupler), and 1 *μ*M rotenone (mitochondrial respiratory complex inhibitors), respectively. (b) Sperm with lower motility exhibited significant decreases in basal OCR, ATP-linked OCR, and maximal OCR. Results are presented as mean ± standard deviation (SD) (*n* = 4) (^∗^*p* < 0.05, ^∗∗^*p* < 0.01, and ^∗∗∗^*p* < 0.001).

Data in Figure 2(a) represent the time course of the OCR analysis under the basal condition, followed by the sequential injections of 5 *μ*M oligomycin (ATP synthase inhibitor), 3 *μ*M FCCP (mitochondrial uncoupler), and 1 *μ*M rotenone (mitochondrial respiratory Complex I inhibitor). The basal, ATP-linked, and maximal OCR were measured in the Percoll-fractionated sperm with and without H_2_O_2_ treatment. The lower basal OCR, ATP-related OCR, and maximal OCR occurred in the sperm with poor motility (Figure 2(b)). Treatment of H_2_O_2_ was found to significantly impair mitochondrial bioenergetics and uncoupled mitochondrial respiration.

### 3.3. Mitochondrial DNA Deletion and 8-OHdG Accumulate in the Sperm with Poor Motility

**Figure 3:**
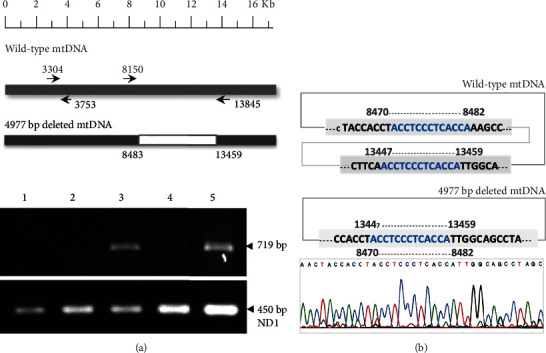
Mitochondrial DNA (mtDNA) deletions in human sperm. (a) An agarose gel electrophoretogram of the PCR products amplified from the mtDNA with the specific 4,977 bp deletion in human sperm using primer-pair L8150-H13845. Lanes 3 and 5 indicate the PCR products of 719 bp amplified from the 4,977 bp deleted mtDNA. Lane 3 was from the infertile subjects with motility scores of 30%. Lane 5 was from the infertile subjects with motility scores of 60%. Lanes 1, 2, and 4 were generated from the normal subjects. The lower gel of the PCR products was amplified from the *ND1* gene using the primer-pair L3304-H3753 for control. (b) Schematic illustration of the nucleotide sequence flanking the junction site at the 5′-end of the 4,977 bp deletion on the heavy strand of the mtDNA in human sperm.

**Figure 4:**
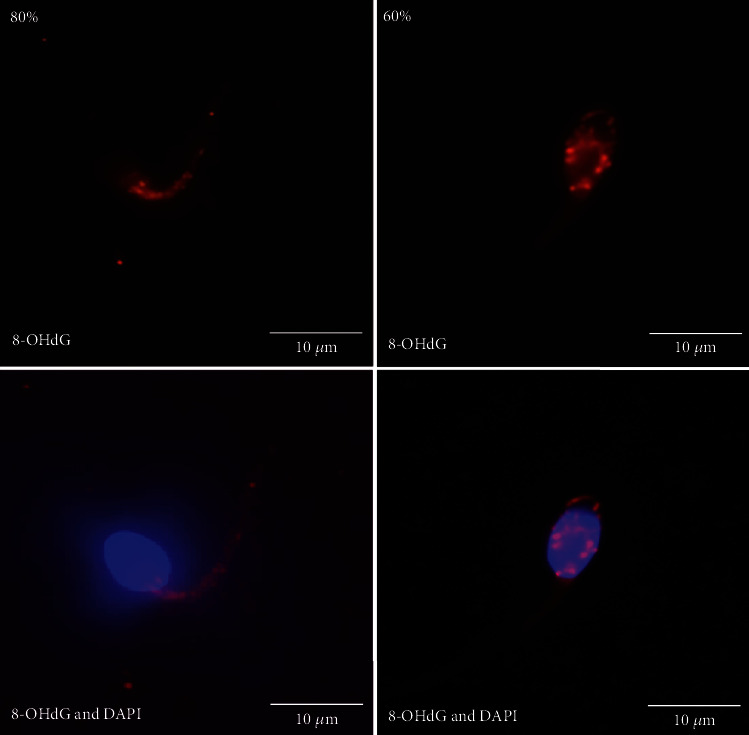
Visualization of 8-OHdG in human sperm. By staining with an anti-8-OHdG antibody conjugated with Alexa Fluor® 647 with the red fluorescent signals, 8-OHdG was identified in the sperm midpiece from the sperm in 80% Percoll gradient. 8-OHdG was found in both the sperm head and midpiece from the sperm in 60% Percoll gradient. The blue fluorescent staining by DAPI was used for labeling the sperm head.

To test whether the mtDNA 4,977 bp deletion accumulates in the sperm with poor motility and infertility, we analyzed the occurrence of the 4,977 bp deletion in 216 sperm samples. We obtained the 719 bp PCR products from the flanking region of the 4,977 bp deleted mtDNA from the individuals with poor sperm motility (Figure 3(a)) and the 450 bp PCR products from the mtDNA *ND1* gene as the mtDNA control. We further identified the nucleotide sequences of the deleted mtDNA in the junction site showing the 13-nucleotide direct repeat (5′-ACCTCCCTCACCA-3′) on the heavy strand of the mtDNA (Figure 3(b)). In addition, we detected the oxidative DNA adducts (8-OHdG) using anti-8-OHdG antibody conjugated with Alexa Fluor® 647 with the red fluorescent signals. The major signals of 8-OHdG were identified in the sperm midpiece from the 80% Percoll gradient. However, 8-OHdG signals were found in both the sperm head and midpiece from the 60% Percoll gradient (Figure 4).

### 3.4. Screening of Gene Polymorphisms Involved in Free Radical Scavenging and Mitochondrial Bioenergetics in Human Sperm

To clarify the associations of sperm capacity and gene mutation (*ATPase6* (m.T8993G) and SNPs of *UCP2* (G-866A, rs659366), *MnSOD* (C47T, rs4880), *CAT* (C-262T, rs1001179), and *hOGG1* (C1245G, rs1052133)), a total of 216 sperm samples were categorized into three groups, i.e., fertile subjects (control group), infertile subjects with more than 50% motility, and infertile subjects with less than 50% motility. The genotype frequencies of *UCP2* G-866A, *MnSOD* C47T, and *CAT* C-262T were found to be significantly different among the fertile subjects (control group), infertile subjects with more than 50% motility, and infertile subjects with less than 50% motility (Table 3). In addition, there were significant differences in the allele frequencies of the *UCP2*-866 G allele (GG and GA, *p* = 0.007) and *MnSOD* 47T allele (TT and TC, *p* = 0.042) among the three groups.

### 3.5. Analyzing the Association of the Occurrence of the MtDNA 4,977 bp Deletion and Gene Polymorphisms Involved in the Free Radical Scavenger System and Mitochondrial Bioenergetics

The impaired sperm motility and fertility group had a higher prevalence of the mtDNA 4,977 bp deletion (Table 4). The higher occurrence of the mtDNA 4,977 bp deletion was found in the sperm of the infertile groups (*p* = 0.047). The occurrence of the mtDNA 4,977 bp deletion was 3.7%, 26.7%, and 16.7% in the fertile subjects (control group), infertile subjects with more than 50% motility, and infertile subjects with less than 50% motility, respectively. In addition, there were significant differences between the occurrence of the mtDNA 4,977 bp deletion and the gene polymorphisms of *MnSOD* (C47T, *p* = 0.042) and *hOGG1* (C1245G, *p* = 0.021; Table 5).

## 4. Discussion

ATP synthesis from the mitochondrial OXPHOS system and glycolysis is essential for human sperm motility [22, 23]. Most studies have concentrated on analyzing mitochondrial respiration to determine whether OXPHOS is crucial for ATP production in sperm [12, 23]. Some studies have shown that sperm motility was inhibited by respiratory inhibitors such as rotenone, potassium cyanide, and oligomycin [24, 25], and stimulated by respiratory substrates (e.g., malate, pyruvate, and lactate) and ADP [26, 27]. These results indicated that proper function of the mitochondrial respiratory enzyme complexes and a tight coupling between respiration and phosphorylation is essential for sperm motility. In addition, a significant decrease in sperm respiratory function was found in asthenozoospermic patients [2]. In addition to its involvement in ATP synthesis, sperm mitochondria may serve as intracellular calcium stores and regulate calcium signaling, ROS signaling, and apoptosis [28]. These observations suggested that mitochondria play a key role in the maintenance of sperm motility and fertility [28, 29].

The mitochondrial membrane potential (ΔΨ*m*) is a potential marker of mitochondrial function and sensitive index of cell damage because it is easily influenced by environmental stress, which is normally associated with the respiratory chain and OXPHOS system. Several studies have substantiated a potential role of ΔΨ*m* in the determination of sperm fertilizability in ejaculated human sperm [30]. The sperm with high ΔΨ*m* represent a subpopulation of sperm with high fertility performance because they have better membrane integrity and higher motility, i.e., they easily undergo a Ca^2+^ ionophore- (A23187-) induced acrosome reaction [30]. In the present study, a significant positive correlation was found between the changes in the mitochondrial membrane potential and human sperm motility. The sperm with better motility were found to have higher ΔΨ*m* (Figures 1). Recently, studies showed that a metabolic assay platform by Seahorse Metabolic Analyzer reveals oxygen consumption rates (OCR) of sperm in real time [31, 32]. Sperm with the best performance had a higher OCR than those that were less motile or immotile [31]. Sperm with higher ratios of oxygen consumption/lactate excretion rate were able to generate higher ATP contents, achieving higher swimming velocities [32]. Comparing to conventional momentary analysis (as in computer-assisted semen analysis), measuring metabolic activity and respiratory capacity of sperm can be an important indicator for sperm quality and their migration success. We found the sperm with higher motility represented higher basal OCR, ATP-linked OCR, and maximal OCR. Here, we found that the treatment with H_2_O_2_ caused dissipation of ΔΨ*m* and bioenergetics in all three sperm groups (Figures 1(b) and 2(b)), suggesting that sperm are susceptible to H_2_O_2_ attack.

Mitochondrial uncoupling is a condition that uncouples proton entry to the mitochondria from ATP synthesis and attenuates the mitochondrial membrane potential. UCPs are a family of inner mitochondrial membrane proteins that are thought to maintain a balance between the energy supply and cell demand in defending cells against ROS production [33, 34]. *UCP2*-866G (rs659366) was found to have higher efficiency of UCP2 expression and promoter activity than -866A. *UCP2* G-866A has been linked to a predisposition to diabetes, obesity, and inflammation [33, 35]. In the present study, the genetic alteration in the *UCP2* G-866A allele was shown to significantly influence sperm fertility and motility.

Upregulation of the enzymes that can neutralize ROS would then be conceivably able to offer at least some protection from the damaging effects. MnSOD converts superoxide to hydrogen peroxide and quenches the free radicals generated by the electron transport chain [36]. A study showed that seminal SOD activity was shown to be positively associated with sperm concentrations and overall motility [37]. Meanwhile, the infertile men with *SOD2* rs4880 CC variants showed a low level of SOD activity compared with that of TT patients [37]. In addition, MnSOD Val16Ala (rs4880) variant genotypes were associated with a significantly higher risk of male infertility [38]. *SOD2* (MnSOD gene) contains the C47T single-nucleotide polymorphism, which results in a Val16Ala amino acid substitution. The C47T results in a valine to alanine substitution in the mitochondrial targeting sequence, leading to an effect on cellular allocation of MnSOD within the mitochondria. The Val allele is partially arrested in the inner mitochondrial membrane, leading to decreased active MnSOD within the mitochondrial matrix [39]. Our results showed that *SOD2* C47T was found to be significantly different among the fertile subjects (control group), infertile subjects with more than 50% motility, and infertile subjects with less than 50% motility.

In addition to MnSOD, catalase contributes to the conversion of H_2_O_2_ to H_2_O and O_2_. A study showed that catalase activities in asthenozoospermic subjects were significantly lower than normozoospermic males [40]. The *CAT* C-262T (rs1001179) polymorphism in the promoter region of the human catalase gene has been associated with lower transcription factor binding and lower catalase expression [41]. In the present study, a higher prevalence of -262T/T and -262C/T genotype frequencies and higher -262T allele frequencies were found in the infertile subjects with less than 50% motility, but without a statistically significant difference among the three groups. This result was consistent with the findings of Sabouhi et al. They showed that the catalase C-262T polymorphism indicates that the CAT -262T/T genotype confers less susceptibility to male infertility [42].

Oxidative stress and related DNA damage in human sperm is important for sperm motility and fertility [10, 43]. The localization of oxidative lesions also differed depending on the genotoxic agent. 8-Hydroxy-2′-deoxyguanosine (8-OHdG) is one of the most abundant oxidative DNA products after H_2_O_2_ treatment [44, 45]. Increased 8-OHdG levels have been identified as influencing pregnancy outcomes [46] and are associated with male pathophysiology such as varicocele [47]. If not repaired, the mutagenic 8-OHdG is associated with DNA fragmentation and may cause structural and functional defects of sperm and may lead to male infertility [11, 15, 48]. Oxidative DNA damage is associated with dysregulation of the acrosome network formation [48] and the impairment of telomere interaction and chromatin condensation [11]. Furthermore, oxidation of the DNA bases in sperm could be a risk factor of de novo mutation transmission to the embryo leading to developmental anomalies and de novo mutations in childhood [15, 43]. The 8-oxoguanine repairs specific enzyme 8-oxoguanine DNA glycosylase (hOGG1) through the base excision repair mechanism. A shift from serine (Ser) to cysteine (Cys) substitution at codon 326, as *hOGG1* C1245G (rs1052133), has been shown to reduce repair activity [49]. The 1245G allele is less effective in repair than the 1245C allele in *hOGG1.* It is known that oxidative damage to mtDNA can cause mitochondrial dysfunction and trigger apoptosis, which may be associated with the accumulation of 8-oxodG. Human OGG1 is also located in the mitochondria (mtOGG1) and has been reported to be associated with mitochondrial function [50]. It has been noted that mtOGG1 suppression was sufficient to diminish mitochondrial respiration and cellular growth rates, and forced expression of mtOGG1 was reversed in those activities [50]. Here, we found that there were no significant differences among the three sperm groups. However, the *OGG1* 1245G allele is associated with the occurrence of the mtDNA 4,977 bp deletion.

In addition to serving as the major intracellular compartment of oxidative metabolism, mitochondria also contain their own genomes. Loss of mtDNA integrity has also been identified in the patients with infertility or subfertility [51–54]. Large-scale deletions of mtDNA have been associated with poor sperm motility [12, 52, 53]. The mtDNA 4,977 bp deletion, also known as mtDNA common deletion, is the most frequent and common mtDNA mutation associated with oxidative damage. In this study, the *SOD2* C47T polymorphism was significantly associated with the occurrence of the mtDNA 4,977 bp deletion. In addition to the mtDNA deletion, male infertility-related single-nucleotide mutations have been reported in eight mtDNA genes, including *ND4*, *COXI*, *COXII*, *COXIII*, *ATPase6*, *ATPase8*, *Cytb*, and *16S rRNA* [55–57]. These single-nucleotide mutations in the mitochondrial genome are associated with poor semen parameters and represent a very important factor affecting sperm maturation, sperm motility, and fertility [58]. In the present study, no association was observed in the mtDNA T8993G mutation among the three sperm groups.

On the basis of our findings, we concluded that mtDNA integrity and energy maintenance may serve as a useful indicator of sperm quality. Our findings also strongly support the hypothesis that the mitochondrial oxidizing microenvironment contributes to the etiopathology of male infertility.

## Figures and Tables

**Table 1:** Characteristics of gene mutations and gene polymorphisms.

Gene	Locus	Reference no.	Position	Function	Characteristics
*UCP2*	G-866A	rs659366	Promoter	Uncoupling proton gradient	Enhanced UCP2 mRNA expression
*MnSOD*	C47T	rs4880	Exon 2	O_2_^•−^⟶H_2_O_2_	Stop translocation of MnSOD at mitochondrial inner membrane, not into matrix
*CAT*	C-262T	rs1001179	Promoter	H_2_O_2_⟶H_2_O	Reduced CAT mRNA expression
*hOGG1*	C1245G	rs1052133	Exon 7	Repair enzyme of 8-OHdG	Reduced hOGG1 enzyme activity
*ATPase6*	T8993G		mtDNA	Respiratory complex	ATP depletion

**Table 2:** Primer sequences and predicted sizes of PCR products in this study.

Gene	Primer sequences	RE	PCR products	PCR-RE products
*UCP2* G-866A	F: 5′-CACGCTGCTTCTGCCAGGAC-3′ R: 5′-AGGCGTCAGGAGATGGACCG-3′	MluI	360 bp	G: 290/70 bp A: 360 bp
*MnSOD* C47T	F: 5′-CAGCCCAGCCTGCGTAGACGG-3′ R: 5′-GCGTTGATGTGAGGTTCCAG-3′	BsaWI	172 bp	T: 88/84 bp C: 172 bp
*CAT* C-262T	F: 5′-AGAGCCTCGCCCCGCCGGACCG-3′ R: 5′-TAAGAGCTGAGAAAGCATAGCT-3′	SmaI	340 bp	C: 185/155 bp T: 340 bp
*hOGG1* C1245G	F: 5′-ACTAGTCTCACCAGCCGTGAC-3′ R: 5′-TGGCCTTTGAGGTAGTCACAG-3′	Fun4HI	293 bp	G: 123/124 bp 169/170 bp C: 293 bp
*ATPase6* T8993G	F: 5′-GACTAATCACCACCCAAC-3′ R: 5′-TGTCGTGCAGGTAGAGGCTT-3′	Ava I	551 bp	T: 551 bp G: 345/206 bp
mtDNA ∆4977	L8150: 5′-CCGGGGGTATACTACGGTCA-3′ H13845: 5′-GTCTAGGGCTGTTAGAAGTC-3′		719 bp	

∆4977: 4977 bp deletion; RE: restriction endonuclease.

**Table 3:** Genotype frequencies of the gene polymorphisms on human male fertility and sperm motility.

Gene	Locus	Reference number	Group		No.	Genotype frequency (%)			*p* value
			Fertility	Motility					
*UCP2*	G-866A	rs659366	Normal		54	GG (46.3)	GA (46.3)	AA (7.4)	0.019
			Infertile	>50%	111	GG (36.9)	GA (38.8)	AA (24.3)	
			Infertile	<50%	51	GG (25.5)	GA (27.4)	AA (47.1)	
*MnSOD*	C47T	rs4880	Normal		54	TT (68.5)	TC (18.5)	CC (13.0)	0.017
			Infertile	>50%	111	TT (41.5)	TC (39.6)	CC (18.9)	
			Infertile	<50%	51	TT (35.3)	TC (39.2)	CC (25.4)	
*CAT*	C-262T	rs1001179	Normal		54	CC (79.6)	TC (13.0)	TT (7.4)	0.091
			Infertile	>50%	111	CC (71.2)	TC (17.1)	TT (11.7)	
			Infertile	<50%	51	CC (62.7)	TC (21.6)	TT (15.7)	
*hOGG1*	C1245G	rs1052133	Normal		54	CC (38.9)	CG (44.4)	GG (16.7)	0.403
			Infertile	>50%	111	CC (32.4)	CG (48.6)	GG (19.0)	
			Infertile	<50%	51	CC (29.4)	CG (49.0)	GG (21.6)	

**Table 4:** Allelic frequencies of the gene polymorphisms and mutation frequencies of mitochondrial DNA on human male fertility and sperm motility.

Gene	Locus	Group		No.	Frequency (%)	*p* value
		Fertility	Motility			
*UCP2*	nDNA	Normal		54	GG + GA (92.6)	0.007
	G-866A	Infertile	>50%	111	GG + GA (75.7)	
		Infertile	<50%	51	GG + GA (52.9)	
*MnSOD*	nDNA	Normal		54	TT + CT (31.5)	0.042
	C47T	Infertile	>50%	111	TT + CT (58.6)	
		Infertile	<50%	51	TT + CT (64.8)	
*CAT*	nDNA	Normal		54	TT + CT (20.4)	0250
	C-262T	Infertile	>50%	111	TT + CT (28.8)	
		Infertile	<50%	51	TT + CT (37.3)	
*hOGG1*	nDNA	Normal		54	GG + CG (61.1)	0.081
	C1245G	Infertile	>50%	111	GG + CG (67.6)	
		Infertile	<50%	51	GG + CG (70.6)	
*ATPase6*	mtDNA	Normal		54	T8993G (1.8)	0.247
	T8993G	Infertile	>50%	111	T8993G (1.8)	
		Infertile	<50%	51	T8993G (1.9)	
∆4977	mtDNA	Normal		54	∆4977 (3.7)	0.047
	np8483-13459	Infertile	>50%	111	∆4977 (24.3)	
		Infertile	<50%	51	∆4977 (17.6)	

nDNA: nuclear DNA; mtDNA: mitochondrial DNA; ∆4977: 4977 bp mtDNA deletion.

**Table 5:** Genotype frequencies of the gene polymorphisms in the mtDNA 4,977 bp deletion.

Gene	Locus	Group	No.	Genotype frequency (%)			*p* value
*UCP2*	G-866A	∆4977(-)	178	GG (41.0)	GA (38.8)	AA (20.2)	0.019
		∆4977(+)	38	GG (15.8)	GA (34.2)	AA (50.0)	
*MnSOD*	C47T	∆4977(-)	178	CC (52.8)	CT (34.3)	TT (12.9)	0.017
		∆4977(+)	38	CC (18.4)	CT (34.2)	TT (47.4)	
*CAT*	C-262T	∆4977(-)	178	CC (74.7)	TC (15.7)	TT (9.6)	0.326
		∆4977(+)	38	CC (55.3)	TC (23.7)	TT (21.0)	
*hOGG1*	C1245G	∆4977(-)	178	CC (39.3)	CG (51.7)	GG (9.0)	0.021
		∆4977(+)	38	CC (5.3)	CG (28.9)	GG (65.8)	

## Data Availability

Data available on request.

## Supplementary Materials

Supplementary Materials Supplementary Table 1: description of patient characteristics.
