# Supplementary material for: Genetic Association in the Maintenance of the Mitochondrial Microenvironment and Sperm Capacity
Source: Oxid Med Cell Longev. 2021 Sep 4;2021:5561395. doi: 10.1155/2021/5561395 (PMC8437596; doi:10.1155/2021/5561395)
Supplement: Supplementary Materials — Supplementary Table 1: description of patient characteristics. [file 5561395.f1.docx]

Supplementary Table 1 Description of patient characteristics.

| **No** | **Age** | **Motility** | **Infertility** | **UCP2** | **MnSOD** | **CAT** | **hOGG1** | **Δ4977** | **ATPase 6** |
| --- | --- | --- | --- | --- | --- | --- | --- | --- | --- |
| S01 | 49 | <50 | M | GA | CC | CC | CC | N | Normal |
| S02 | 48 | <50 | M | GA | CC | CC | CG | N | Normal |
| S05 | 12 | <50 | M | GA | CC | CC | CG | N | Normal |
| S06 | 60 | >50 | M | GA | CC | CC | CG | N | Normal |
| S07 | 55 | >50 | M | GA | TT | CC | CG | N | Normal |
| S12 | 5 | <50 | M | AA | CC | CT | CC | N | Normal |
| S13 | 81 | >50 | M | GG | CC | CC | CG | N | Normal |
| S16 | 69 | >50 | M | GA | TT | TT | CC | N | Normal |
| S17 | 7 | <50 | M | AA | CC | CC | GG | P | Normal |
| S18 | 86 | >50 | M | AA | CT | CC | CC | N | Normal |
| S19 | 38 | <50 | M | AA | TT | CC | CC | N | Normal |
| S20 | 53 | >50 | M | GA | CT | CC | CC | N | Normal |
| S21 | 46 | <50 | M | AA | CT | CC | CG | N | Normal |
| S22 | 65 | >50 | M | AA | TT | CC | GG | P | Normal |
| S25 | 45 | <50 | M | AA | CC | TT | CC | P | Normal |
| S26 | 35 | <50 | M | GG | CT | CC | CG | N | Normal |
| S27 | 80 | >50 | M | GA | CC | CC | CC | N | Normal |
| S28 | 27 | <50 | M | AA | TT | CC | GG | P | Normal |
| S29 | 72 | >50 | M | GA | TT | CC | CC | N | Normal |
| S32 | 2 | <50 | M | AA | CT | CT | CG | N | Normal |
| S33 | 60 | >50 | M | GA | CC | CC | CG | N | T8993G |
| S35 | 63 | >50 | M | GG | CT | CC | CG | N | Normal |
| S36 | 51 | >50 | M | AA | CT | CC | GG | P | Normal |
| S37 | 5 | <50 | M | GA | CT | CC | CC | N | Normal |
| S39 | 72 | >50 | M | AA | TT | CC | CG | P | Normal |
| S40 | 18 | <50 | M | GA | CT | CC | CC | N | Normal |
| S42 | 58 | >50 | M | GG | CT | TT | CG | N | Normal |
| S43 | 35 | <50 | M | GA | CT | CC | CC | N | Normal |
| S44 | 70 | >50 | M | GG | CT | CC | GG | P | Normal |
| S45 | 57 | >50 | M | GA | TT | CT | GG | P | Normal |
| S46 | 41 | <50 | M | GG | CT | CC | CC | N | Normal |
| S47 | 60 | >50 | M | GA | CT | CT | GG | P | Normal |
| S48 | 86 | >50 | M | GA | CT | CT | CG | P | Normal |
| S49 | 71 | >50 | M | AA | CC | CC | CG | N | Normal |
| S50 | 66 | control | F | GA | CC | CC | CG | N | Normal |
| S51 | 78 | control | F | GA | CT | CC | CC | N | Normal |
| S52 | 90 | control | F | GG | CT | CC | CG | N | Normal |
| S53 | 92 | >50 | M | AA | CT | CT | CG | p | Normal |
| S54 | 25 | <50 | M | AA | TT | CC | GG | p | Normal |
| S55 | 65 | >50 | M | GA | CT | TT | CG | N | Normal |
| S56 | 52 | >50 | M | GG | CT | TT | CC | N | Normal |
| S57 | 70 | >50 | M | AA | CC | CC | CC | N | Normal |
| S58 | 20 | <50 | M | AA | CT | CT | CG | N | T8993G |
| S59 | 53 | >50 | M | GG | TT | CT | CC | N | Normal |
| S60 | 82 | >50 | M | GG | CT | CC | CG | N | Normal |
| S61 | 74 | >50 | M | AA | CC | CC | CG | N | Normal |
| S62 | 6 | <50 | M | GA | CC | CC | CG | N | Normal |
| S63 | 45 | <50 | M | GG | CC | CT | CG | N | Normal |
| S64 | 55 | >50 | M | AA | CT | CT | CG | N | Normal |
| S65 | 88 | >50 | M | GA | CC | CC | CG | N | Normal |
| S66 | 20 | <50 | M | AA | CC | CC | CC | N | Normal |
| S67 | 59 | >50 | M | GG | CT | CC | GG | P | Normal |
| S68 | 80 | control | F | GA | CT | CC | CG | N | Normal |
| S69 | 92 | >50 | M | AA | CC | CT | CG | N | Normal |
| S70 | 70 | >50 | M | GG | CT | CC | GG | N | Normal |
| S71 | 68 | >50 | M | AA | CC | CC | CC | N | Normal |
| S72 | 80 | >50 | M | GA | CT | CC | CG | P | Normal |
| S73 | 90 | control | F | GA | CC | CC | CG | N | Normal |
| S78 | 0 | <50 | M | GA | TT | CT | GG | P | Normal |
| S79 | 0 | <50 | M | GG | CC | CT | GG | N | Normal |
| S80 | 78 | >50 | M | GG | CT | CC | CG | N | Normal |
| S81 | 55 | >50 | M | GG | CT | CT | GG | P | Normal |
| S82 | 66 | >50 | M | GG | CC | CC | CG | N | Normal |
| S83 | 74 | >50 | M | GG | CT | CC | CC | N | Normal |
| S84 | 40 | control | F | GA | CC | CC | CG | N | Normal |
| S85 | 82 | >50 | M | GG | CC | CC | CG | N | Normal |
| S86 | 80 | >50 | M | GA | CT | CC | CG | N | Normal |
| S87 | 55 | >50 | M | GG | CC | CC | CG | N | Normal |
| S88 | 0 | <50 | M | AA | CT | CC | CG | N | Normal |
| S89 | 77 | >50 | M | GG | CC | CC | CC | N | Normal |
| S90 | 87 | >50 | M | GG | CC | CC | GG | N | Normal |
| S91 | 65 | >50 | M | GG | CC | CC | CG | N | Normal |
| S92 | 36 | >50 | M | AA | CC | CC | CG | N | Normal |
| S93 | 79 | >50 | M | GG | CC | CC | CG | N | Normal |
| S94 | 0 | <50 | M | AA | TT | CT | GG | P | Normal |
| S95 | 82 | >50 | M | AA | CC | CC | CG | N | Normal |
| S96 | 53 | >50 | M | GG | CT | CT | CG | N | Normal |
| S97 | 0 | <50 | M | GA | CT | TT | GG | N | Normal |
| S98 | 88 | >50 | M | AA | CT | CC | CC | N | Normal |
| S99 | 34 | <50 | M | GG | TT | CT | GG | N | Normal |
| S100 | 64 | >50 | M | GG | CC | CC | CG | N | Normal |
| S101 | 40 | control | F | AA | CC | TT | GG | N | Normal |
| S102 | 40 | <50 | M | GG | CC | CT | CC | N | Normal |
| S103 | 60 | >50 | M | AA | CC | CC | CC | N | Normal |
| S104 | 76 | >50 | M | GA | CT | CC | GG | P | Normal |
| S105 | 66 | >50 | M | GG | CT | TT | CC | N | Normal |
| S106 | 72 | >50 | M | GG | CT | TT | CC | N | Normal |
| S107 | 59 | >50 | M | GA | CC | CC | GG | P | Normal |
| S108 | 30 | control | F | AA | CC | TT | GG | P | Normal |
| S109 | 86 | >50 | M | GG | TT | CC | CG | P | Normal |
| S110 | 40 | <50 | M | GG | CT | CC | CG | N | Normal |
| S111 | 15 | control | F | GG | CC | TT | CG | N | Normal |
| S112 | 77 | >50 | M | GA | TT | CC | GG | P | Normal |
| S113 | 73 | >50 | M | AA | TT | CC | CG | P | Normal |
| S114 | 72 | control | F | GA | CT | CC | CC | N | Normal |
| S115 | 32 | <50 | M | GG | CT | CC | CG | N | Normal |
| S116 | 92 | control | F | GA | CT | CC | GG | N | Normal |
| S117 | 80 | >50 | M | GG | CC | CC | CC | N | Normal |
| S118 | 77 | >50 | M | GA | CT | CC | CC | N | Normal |
| S119 | 53 | >50 | M | GG | CT | CT | CC | N | Normal |
| S120 | 80 | >50 | M | GG | CT | CC | CC | N | Normal |
| S121 | 80 | >50 | M | GG | CC | CC | CG | N | Normal |
| S122 | 65 | >50 | M | GG | CT | CC | CC | N | Normal |
| S123 | 90 | control | F | GA | TT | CC | CC | N | Normal |
| S124 | 54 | control | F | GA | CC | CC | CC | N | Normal |
| S125 | 79 | control | F | GA | CC | CC | CG | N | Normal |
| S126 | 0 | <50 | M | GA | TT | TT | CG | N | Normal |
| S127 | 80 | >50 | M | GA | CT | CC | CC | N | Normal |
| S128 | 68 | >50 | M | GG | CC | CT | CC | N | Normal |
| S129 | 88 | >50 | M | GG | CC | CC | CC | N | Normal |
| S130 | 72 | >50 | M | GA | CC | CC | CC | N | Normal |
| S131 | 80 | >50 | M | GG | CC | CT | CG | N | Normal |
| S132 | 88 | >50 | M | GA | CC | CC | CC | N | Normal |
| S133 | 46 | <50 | M | GA | TT | TT | GG | P | Normal |
| S134 | 66 | control | F | GA | TT | CC | CG | N | Normal |
| S135 | 80 | >50 | M | AA | CT | TT | GG | P | Normal |
| S136 | 85 | control | F | GA | TT | CC | GG | N | Normal |
| S137 | 77 | control | F | GA | CC | CC | CC | N | Normal |
| S138 | 69 | control | F | GG | TT | CC | CG | N | Normal |
| S139 | 81 | control | F | GA | TT | CC | GG | N | Normal |
| S140 | 88 | control | F | GG | CT | CC | CC | N | Normal |
| S141 | 72 | control | F | GG | CT | CT | CC | N | Normal |
| S142 | 82 | >50 | M | AA | CC | TT | GG | P | Normal |
| S143 | 92 | >50 | M | GG | CT | CC | CG | P | Normal |
| S144 | 68 | >50 | M | GA | CC | CC | CG | N | Normal |
| S145 | 72 | >50 | M | AA | TT | CT | CG | P | Normal |
| S146 | 64 | control | F | GG | CC | CC | CC | N | Normal |
| S147 | 83 | control | F | AA | CC | CC | CG | N | Normal |
| S148 | 74 | control | F | GG | CC | CC | CG | N | Normal |
| S149 | 25 | <50 | M | AA | CC | TT | CC | N | Normal |
| S150 | 70 | control | F | GG | CC | CC | CG | N | Normal |
| S151 | 21 | <50 | M | GG | TT | CC | CG | N | Normal |
| S152 | 64 | control | F | AA | CC | CC | CG | N | Normal |
| S153 | 45 | <50 | M | GA | TT | CC | CG | N | Normal |
| S154 | 57 | >50 | M | GA | CC | CC | CC | N | Normal |
| S155 | 57 | control | F | GG | CC | CT | CG | N | Normal |
| S156 | 64 | control | F | GG | TT | CC | CC | N | Normal |
| S157 | 41 | <50 | M | GG | CT | CT | CC | N | Normal |
| S158 | 85 | control | F | GA | CC | CC | CG | N | Normal |
| S159 | 75 | control | F | GA | CC | CT | CG | N | Normal |
| S160 | 75 | >50 | M | GG | TT | TT | CG | P | Normal |
| S161 | 47 | <50 | M | AA | CT | CC | CC | N | Normal |
| S162 | 51 | >50 | M | GA | TT | CT | CC | N | Normal |
| S163 | 48 | <50 | M | GA | CC | CC | CG | N | Normal |
| S164 | 70 | control | F | GA | TT | CT | CG | N | Normal |
| S165 | 46 | <50 | M | GA | CT | CC | CG | N | Normal |
| S166 | 48 | <50 | M | AA | CT | CC | CG | N | Normal |
| S167 | 64 | >50 | M | GA | TT | CC | GG | P | Normal |
| S168 | 80 | >50 | M | GA | CT | CC | CG | N | Normal |
| S169 | 59 | >50 | M | AA | CT | TT | CG | N | Normal |
| S170 | 68 | control | F | GG | CT | CT | CG | N | Normal |
| S171 | 75 | control | F | GA | CC | CT | GG | P | Normal |
| S172 | 78 | control | F | GA | CC | CC | CC | N | Normal |
| S173 | 43 | <50 | M | AA | TT | CC | CG | N | Normal |
| S174 | 66 | >50 | M | GA | CC | CC | CC | N | Normal |
| S175 | 51 | control | F | GA | CC | CC | CG | N | Normal |
| S176 | 65 | >50 | M | GA | TT | CC | GG | P | Normal |
| S177 | 65 | >50 | M | GA | CT | CC | CC | N | Normal |
| S178 | 80 | >50 | M | GA | CC | TT | CG | P | Normal |
| S179 | 59 | >50 | M | GA | CC | CC | CG | N | Normal |
| S180 | 63 | >50 | M | GA | CC | CC | GG | N | Normal |
| S181 | 59 | control | F | GG | CC | CC | CC | N | Normal |
| S182 | 65 | >50 | M | GG | CC | CC | CG | N | Normal |
| S183 | 42 | <50 | M | AA | CC | CT | CG | N | Normal |
| S184 | 55 | >50 | M | GA | CC | CC | CG | N | Normal |
| S185 | 65 | >50 | M | GA | TT | CC | CC | N | Normal |
| S186 | 60 | >50 | M | GA | CC | CT | CC | N | Normal |
| S187 | 47 | <50 | M | GG | CC | CC | CG | N | Normal |
| S188 | 72 | >50 | M | GA | CT | CC | CC | N | Normal |
| S189 | 70 | control | F | GG | CC | CC | GG | N | Normal |
| S190 | 74 | >50 | M | GG | CC | CT | GG | N | Normal |
| S191 | 80 | >50 | M | AA | CC | CC | CG | N | Normal |
| S192 | 58 | >50 | M | GA | TT | CC | CC | N | Normal |
| S193 | 66 | >50 | M | GA | CT | CC | GG | N | Normal |
| S194 | 57 | >50 | M | GG | CC | CC | CC | N | Normal |
| S195 | 60 | >50 | M | GA | CC | CT | CG | N | Normal |
| S196 | 62 | control | F | GA | CT | CC | CC | N | Normal |
| S197 | 54 | control | F | GG | CC | CC | CG | N | Normal |
| S198 | 55 | >50 | M | AA | CT | CC | CG | N | Normal |
| S199 | 60 | >50 | M | GG | CT | CT | CG | N | Normal |
| S200 | 47 | <50 | M | AA | CT | CC | CC | N | Normal |
| S201 | 60 | >50 | M | AA | CT | CC | CG | N | Normal |
| S202 | 75 | >50 | M | GG | TT | CC | CC | N | Normal |
| S203 | 75 | control | F | GA | CC | TT | GG | N | Normal |
| S204 | 65 | >50 | M | GG | TT | TT | CG | N | Normal |
| S205 | 53 | >50 | M | GG | CT | CC | CG | N | Normal |
| S206 | 64 | >50 | M | AA | CT | CC | GG | P | Normal |
| S207 | 58 | control | F | GA | CC | CC | CG | N | Normal |
| S208 | 76 | >50 | M | AA | CT | CC | CG | P | Normal |
| S209 | 37 | <50 | M | GG | CT | CC | CG | N | Normal |
| S210 | 72 | control | F | GG | CC | CC | CC | N | Normal |
| S211 | 48 | <50 | M | AA | CC | CC | GG | N | Normal |
| S212 | 56 | >50 | M | AA | TT | CC | GG | P | Normal |
| S213 | 43 | <50 | M | AA | CT | CC | CG | N | Normal |
| S214 | 52 | control | F | GA | CC | CC | CC | N | Normal |
| S215 | 74 | control | F | GG | CC | CT | CC | N | Normal |
| S216 | 30 | <50 | M | AA | CC | TT | CG | N | Normal |
| S217 | 80 | >50 | M | GA | CT | CC | CC | N | Normal |
| S218 | 65 | >50 | M | GA | CC | CC | CG | N | Normal |
| S219 | 32 | <50 | M | GG | CC | CC | CG | N | Normal |
| S220 | 41 | <50 | M | AA | TT | TT | GG | P | Normal |
| S221 | 16 | <50 | M | AA | TT | CC | CC | P | Normal |
| S222 | 38 | <50 | M | AA | CT | TT | CG | N | Normal |
| S223 | 62 | >50 | M | AA | TT | CT | CG | N | Normal |
| S224 | 72 | >50 | M | GA | CC | TT | CG | N | T8993G |
| S225 | 69 | >50 | M | GA | CC | CC | CG | N | Normal |
| S226 | 72 | control | F | GG | CT | CC | CC | N | Normal |
| S227 | 63 | control | F | GG | CC | CC | GG | N | Normal |
| S228 | 81 | control | F | GG | CC | CC | CC | N | Normal |
| S229 | 82 | control | F | GA | CC | CC | CC | N | Normal |
| S230 | 67 | control | F | GG | CC | CC | CC | N | Normal |
| S231 | 53 | control | F | GG | CC | CC | CC | N | Normal |
| S232 | 76 | control | F | GG | CC | CC | CC | N | Normal |
| S233 | 71 | control | F | GG | CC | CC | CG | N | Normal |
| S234 | 77 | control | F | GG | CC | CC | CG | N | Normal |
| S235 | 88 | control | F | GG | CC | CC | CG | N | T8993G |
